# Removal of a temporal lobe cavernous angioma to control epileptic seizures in a patient with tuberous sclerosis complex

**DOI:** 10.1016/j.heliyon.2020.e04229

**Published:** 2020-06-23

**Authors:** Kazuki Sakakura, Ayataka Fujimoto, Naoki Ichikawa, Shimpei Baba, Hideo Enoki, Tohru Okanishi

**Affiliations:** Comprehensive Epilepsy Center, Seirei Hamamatsu General Hospital, Japan

**Keywords:** Tuberous sclerosis complex, Cavernous angioma, Intraoperative electro-corticography, Concomitance, Neurology, Surgery, Pediatrics, Medical imaging, Diagnostics

## Abstract

**Purpose:**

A patient with tuberous sclerosis complex (TSC) and a left temporal cavernous angioma (CA) presented with treatment-resistant epilepsy. We evaluated the patient to determine the best treatment option.

**Patient and methods:**

A 7-year-old boy with TSC exhibited weekly impaired awareness seizures and was diagnosed with TSC based on the modified Gomez's criteria. The presence of cortical tubers had been noted by his physicians. However, left temporal CA had not been diagnosed. He was referred to our facility for further treatment at the age of 33. Presurgical evaluation in our facility revealed the brain tubers and left temporal CA. Based on his seizure semiology, magnetic resonance imaging, scalp electroencephalogram, and long-term video monitoring, we determined his seizures were from the CA and not the TSC network. We then performed intraoperative-electrocorticography (ECoG).

**Results:**

Because the ECoG showed epileptiform discharges from the surrounding area of the CA but not from other areas, we removed the CA. He has been seizure-free for more than 10 years.

**Conclusion:**

The higher likelihood of TSC as well as greater familiarity with this disorder might lead physicians to overlook the possibility of CA.

## 1 Introduction

The occurrence rate of epilepsy in the tuberous sclerosis complex (TSC) is 72–85% [1], and 63% of cases are treatment-resistant [2]. The occurrence rate of epilepsy in a patient with a cavernous angioma (CA) is 32–51% [3], and 78% of cases are treatment-resistant [4]. As there have been no reports of TSC and CA occurring concomitantly, it is not known whether TSC, CA, or both together are responsible for epileptic seizures. Because the occurrence rate and intractability rate of epilepsy are similar, we had difficulty planning a treatment strategy for a patient with treatment-resistant epilepsy who simultaneously had TSC and a temporal lobe CA. Here, we present a patient with TSC who presented with focal impaired awareness seizures (FIAS) and was initially diagnosed with TSC-associated epilepsy as a child.

### 1.1 Ethics approval

Submission of this case report was approved by the ethics review board at Seirei General Hospital, and written informed consent was obtained from the patient.

## 2 Patient and methods

**Figure 1:**
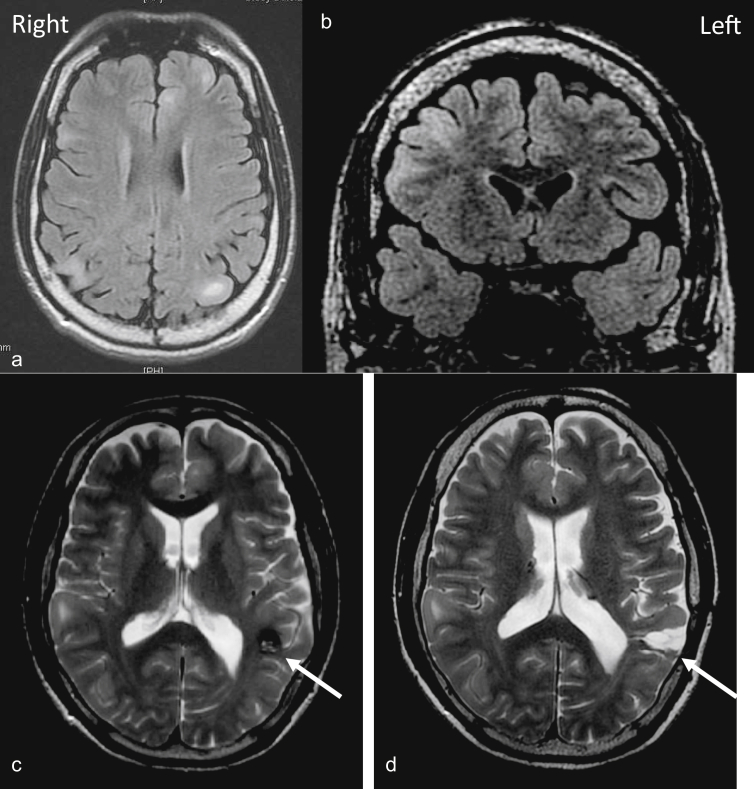
Multiple cortical tubers and pre-/post-surgical magnetic resonance image (MRI). The fluid-attenuated inversion recovery (FLAIR) MRI shows multiple tubers (a and b). The T2-weighted MRI shows the pre- (c) and post- (d) removal of the CA (arrow).

**Figure 2:**
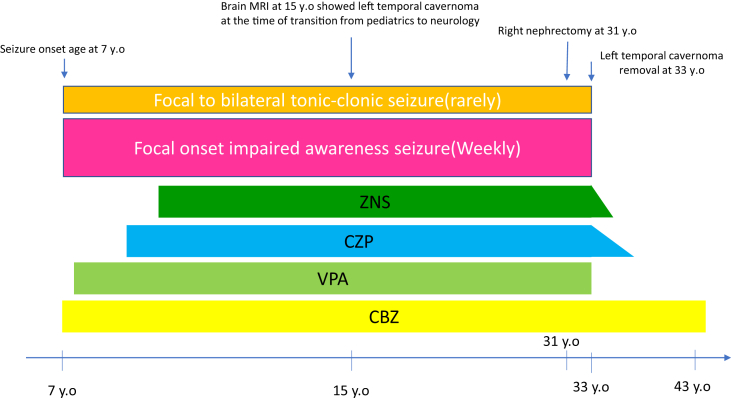
Clinical timeline of the patient. He exhibited focal onset impaired awareness seizure at the age of 7. He had been treated with carbamazepine (CBZ), valproate (VPA), zonisamide (ZNS), and clonazepam (CZP) over the years. The left temporal cavernoma removal was performed at the age of 33.

A 7-year-old boy presented with FIAS, consisting of drooling and staring with loss of awareness, preceded by right facial twitching. FIAS lasted around 1–2 min and occurred weekly. Seizures rarely became bilateral tonic-clonic seizures. He had multiple cortical tubers in his brain, along with bilateral renal angiomyolipomas, facial angiofibromas, shagreen patches, and ash leaf patches, and was diagnosed with TSC based on the modified Gomez's criteria. His parents underwent dermatological and radiological examinations, but no findings compatible with TSC were found. Neither he nor his family wanted genetic testing, so this examination was not performed. He received anti-seizure medication (ASM) therapy with carbamazepine, valproate, zonisamide, and clonazepam. At age 31, he underwent a right nephrectomy due to life-threatening retroperitoneal hemorrhage. At age 33, he was referred to our facility because his seizures had not been controlled by the ASMs. In the referral letters, one of the reports written by a radiologist about his brain magnetic resonance imaging (MRI) findings at the time of transition from the department of pediatrics to adult neurology (at age 15) said that there was a CA in the left temporal lobe. However, it seems likely that his adult neurologist did not read this report. He underwent another brain MRI at our facility, and findings showed multiple tubers and a left temporal CA (Figure 1). A scalp electroencephalogram (EEG) showed intermittent medium amplitude epileptiform discharges over the left posterior head region. Based on the seizure semiology, the MRI, and EEG findings, we considered that the CA on the left temporal lobe might be responsible for his seizures. He then underwent pre-surgical evaluation including long-term video EEG (VEEG), the Wada test, and neuropsychological examinations. Figure 2 shows the clinical timeline of the patient.

## 3 Results

The VEEG showed ictal activity arising from the left posterior temporal area with stereotypical FIAS. His Wechsler Adult Intelligence Scale, third edition, showed a verbal intelligent quotient (IQ) of 67, performance IQ of 82, and full IQ of 72. The Wada test showed a left language-dominant hemisphere.

**Figure 3:**
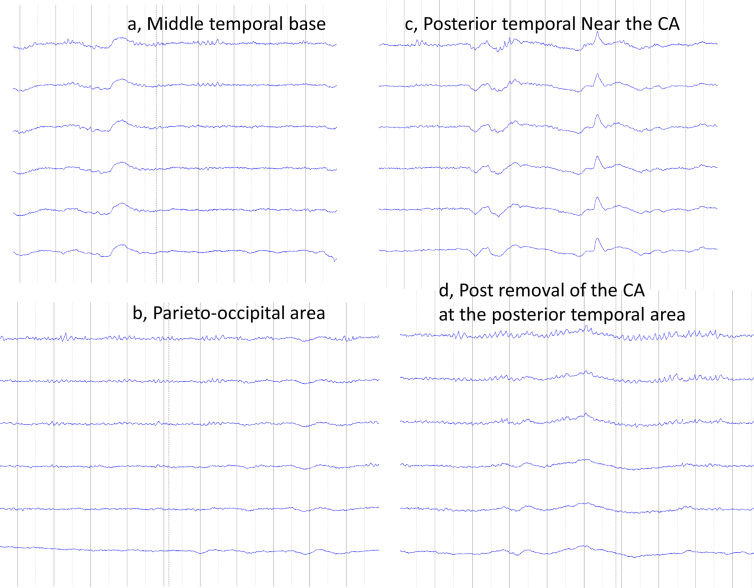
Intra-operative electrocorticography (ECoG). The middle temporal base-ECoG shows slow waves, probably from the cavernous angioma (CA) (a). The ECoG from the parieto-occipital area showed no epileptiform discharges. The CA was removed under the awake state because the Wada test showed a left language-dominant hemisphere, and occipital alpha activity was seen (b). Epileptiform discharges were observed near the CA (c). Postoperatively, no epileptiform discharges were seen (d).

Because 1) the ictal scalp EEG showed ictal activity arising from left posterior temporal region; 2) the location of the CA was concordant with the ictal scalp EEG findings; 3) his verbal IQ was more impaired than his performance IQ; and 4) no cortical tubers were seen in the left posterior temporal area, we regarded his seizures as coming from the CA and not from the TSC-associated epilepsy network in his brain. We performed intraoperative electrocorticography (ECoG), which showed epileptiform discharges only from the contacts near the CA (Figure 3). Based on these findings, we removed the CA during an awake surgery because his language dominant hemisphere was on the left side. After removal of the CA, the ECoG near the area of the CA removal showed no epileptiform discharges. More than 10 years have passed since the CA removal. He rarely experiences psychogenic non-epileptic spells, and these spells are completely different from the FIAS and may last more than 1 h. He has been seizure-free for 10 years without any neurological deficits. He had been on Carbamazepine after the surgery.

## 4 Discussion

From this case, we found that:

1) Epileptic seizures starting at the CA and moving to the TSC were not present.
2) The higher likelihood of TSC as well as a greater familiarity with this disorder might lead physicians to overlook the possibility of CA.

Gene abnormalities have been identified in the pathology of both TSC and CA such as *TSC1 TSC2* genes in the TSC [5], and *CCM1, CCM2,* and *CCM3* genes in a CA [6]. However, to the best of our knowledge, no reports of concomitant TSC and CA have been published, and thus the diagnosis of this patient could not be based on a combination of gene abnormalities [7] or the concomitant occurrence of these conditions. In other words, we cannot be sure whether the concomitant TSC and CA happened by chance or via combined genetic abnormalities.

TSC treatment requires a well-organized interdisciplinary team [8, 9]. However, in real-world practice in Japan, these well-organized team have not been established. Additionally, there are issues when patients transition from pediatrics to adult practices [10]. This was also likely one of the reasons the CA in this patient was overlooked for more than 25 years, and he experienced weekly uncontrollable seizures from age 7. Because the ECoG showed epileptiform discharges, and removal of CA resulted in an absence of seizures for more than 10 years, it seems clear that the CA was responsible for the seizures. In addition, the lack of response to ASMs also indicated that the TSC may not have caused his seizures. One paper reported that 33.5% of patients with TSC became seizure-free with ASMs, and 37.5% of them were able to wean off ASMs [11]. The average age at becoming seizure-free was 13 years [11].

## 5 Conclusion

The higher likelihood of TSC as well as greater familiarity with this disorder might lead physicians to overlook the possibility of CA.

## Declarations

### Author contribution statement

All authors listed have significantly contributed to the investigation, development and writing of this article.

### Funding statement

This research did not receive any specific grant from funding agencies in the public, commercial, or not-for-profit sectors.

### Competing interest statement

The authors declare no conflict of interest.

### Additional information

No additional information is available for this paper.
